# Cartographie, profil et formation des acteurs de la vaccination, Bénin, 2020

**DOI:** 10.48327/mtsi.v3i2.2023.349

**Published:** 2023-04-11

**Authors:** Aristide APLOGAN, Didier AGOSSADOU, Dramane PALENFO, Téné-Alima ESSOH, Landry KAUCLEY, Alain Komi AHAWO

**Affiliations:** 1 Agence de médecine préventive Afrique, 08 BP 660 Abidjan 08, Côte d'Ivoire; 2 Agence de médecine préventive Bénin, 03 BP 2309 Cotonou, Bénin; 3 Agence nationale des soins de santé primaires, Ministère de la santé, 01 BP 882, Cotonou, Bénin; 4 Gavi l'Alliance du vaccin, Secrétariat, Genève, Suisse

**Keywords:** Cartographie, Profil, Acteurs, Vaccination, Bénin, Afrique subsaharienne, Mapping, Profile, Stakeholders, Immunization, Benin, Sub-Saharan Africa

## Abstract

**Introduction:**

La Direction de la vaccination et logistique du Bénin a commandité cette étude afin de disposer de données factuelles pour améliorer la performance du Programme élargi de vaccination. Les objectifs étaient de recenser les acteurs de la vaccination, décrire leur profil, leur répartition géographique et déterminer leurs besoins de formation.

**Méthodes:**

En décembre 2020, nous avons recensé les acteurs de la vaccination via leurs dossiers administratifs et des entretiens téléphoniques. Les données étaient collectées dans chaque direction départementale et zone sanitaire par des points focaux à l'aide d'une grille Excel^®^.

**Résultats:**

Nous avons recensé 3 893 acteurs de la vaccination, âgés en moyenne de 39 ans dont 66% de femmes, 53% d'aides-soignants, 77% de vaccinateurs et 56% d'agents contractuels de l’État. Environ 96% d'entre eux exerçaient dans les centres périphériques, 56% n’étaient pas formés et 14% l’étaient depuis plus de 5 ans. Plus de la moitié des acteurs n’étaient pas formés pour leur fonction. Nous avons estimé à 70% la proportion d'acteurs à former, alors que 98% des acteurs souhaitaient être formés notamment en vaccination (74%), gestion des vaccins (69%), chaîne du froid (61%), surveillance des maladies (61%) et maintenance (47%).

**Conclusion:**

La prédominance des aides-soignants et des femmes et la faible proportion de formés parmi les acteurs de la vaccination, étaient également observées dans d'autres pays d'Afrique subsaharienne. Le Bénin devra mobiliser des ressources suffisantes pour renforcer les capacités techniques des acteurs de la vaccination.

## Abstract (en)

**Introduction:**

The Benin Immunization and Logistic Directorate commissioned this study to provide evidence-based data to improve the performance of the Expanded Programme on Immunization. The objectives were to identify immunization stakeholders, describe their profile, their geographical distribution and determine their training needs.

**Methods:**

In December 2020, we conducted a census of Benin's immunization stakeholders via their administrative files and telephone interviews. The data were collected in each departmental directorate and health zone by focal points using an Excel^®^ grid. The authorizations of the ethics and personal data protection committees were obtained.

**Results:**

We identified 3, 893 immunization workers, 39 years old on average, 66% of whom were women, 53% were health care assistants, 77% were vaccinators, and 56% were government contract workers. About 96% of them worked in peripheral centers. Approximately 56% were untrained and 14% had been untrained for more than 5 years. More than half of the actors were not trained for their function. The proportion of actors needing training was estimated at 70%. The training needs were at least 72% in the departmental hospitals, zone hospitals and health centers. 98% of actors reported a need for training, including immunization (74%), vaccine management (69%), cold chain (61%), monitoring (61%), and maintenance (47%).

**Conclusion:**

The predominance of nurses’ aides and women and the low proportion of trained personnel among immunization workers were also observed in other sub-Saharan African countries. Benin's Immunization and Logistics Directorate should mobilize sufficient resources to strengthen the technical capacities of the majority of immunization stakeholders.

## Introduction

Le Bénin couvre une superficie de 114 763 km^2^ et comptait en 2021 une population de 13 301 694 habitants. Il comporte 12 directions départementales de la santé (DDS), 34 zones sanitaires (ZS) et 85 communes. Le Programme élargi de vaccination (PEV) y est mis en œuvre depuis 1982 avec une organisation de type pyramidal. Sa gestion est assurée au niveau central par la Direction de la vaccination et logistique (DVL), au niveau départemental par le service de la santé publique et de la médecine traditionnelle et au niveau de la ZS par l’équipe d'encadrement de zone. L'insuffisance quantitative et qualitative des acteurs de vaccination et leur inégale répartition géographique demeurent un problème majeur [1, 5]. Or pour que le PEV soit performant, il lui faut des acteurs formés, motivés et équitablement répartis. La présente étude avait pour objectif de décrire les acteurs de la vaccination selon la catégorie socioprofessionnelle, la formation reçue, le besoin de formation et ceci par niveau de la pyramide sanitaire, par type de structure de soins et par fonction au sein du PEV. L’étude permettra à la Direction de la vaccination et logistique de disposer de données factuelles pour planifier des interventions adaptées pour améliorer les performances du PEV.

## Méthodes

### Cible et échantillonnage

Nous avons recensé les acteurs de la vaccination à tous les niveaux du système de santé.

### Méthodes de collecte et d'analyse des données

C’était une enquête transversale réalisée en décembre 2020 par des points focaux identifiés dans chaque direction départementale de la santé et chaque zone sanitaire. Ces points focaux avaient interrogé par téléphone les acteurs de la vaccination, à l'aide de grilles électroniques d'entretien. Le traitement des données sur Excel avait porté sur l’âge, le sexe, le lieu d'exercice, la catégorie professionnelle, le statut administratif, la fonction au sein du PEV, la formation reçue sur le PEV et la proportion d'acteurs ayant besoin de formation sur le PEV. Cette dernière variable était estimée en additionnant la proportion des acteurs non formés à celle des acteurs formés depuis 5 ans et plus. Ces variables étaient analysées pour le pays, par niveau du système de santé, par type de structure de soin et par fonction au sein du PEV.

#### Considérations éthiques et confidentialité

Conformément à l'article 389 de la loi n° 2017-20 du 20 avril 2018 portant code du numérique en République du Bénin, le traitement des données à caractère personnel est légitime si la personne concernée donne son consentement écrit. Il peut être dérogé à cette exigence lorsque le traitement est nécessaire à l'exécution d'une mission d'intérêt public. Néanmoins, l'autorisation de l'autorité de protection des données personnelles et celle du comité local d’éthique pour la recherche biomédicale de l'université de Parakou [8] ont été obtenues. Nous avons également recueilli le consentement oral de tous les acteurs de la vaccination avant les entretiens.

## Résultats

L’étude a permis de constituer une base de données électronique de tous les acteurs de la vaccination du Bénin, y compris leurs localisations géographiques. Le taux de réponse était de 100% car tous les acteurs recensés ont répondu. Mais il faut signaler qu'il y a eu quelques données manquantes pour certaines questions non essentielles.

### Profil et formation des acteurs pour le pays et par niveau du système de santé

En décembre 2020, nous avons recensé au Bénin 3 893 acteurs de la vaccination âgés de 39 ans en moyenne (Tableau I). Dans les centres périphériques, la majorité des acteurs étaient des vaccinateurs (76%) puis des aides-soignants (49%). À tous les niveaux sauf à la Direction de la vaccination et logistique, plus de la moitié des acteurs de la vaccination étaient des contractuels de l’État.

**Tableau I:** Répartition et profil des acteurs de la vaccination par niveau du système de santé, Bénin, 2020 Distribution and profile of immunization stakeholders according to level in the health system, Benin, 2020

		DVL	DDS	BZS	Centres périphériques	Total
		(n = 10)	(n = 68)	(n = 92)	(n = 3723)	(n = 3893)
**Âge (ans)**	minimum	36	32	25	18	18
	maximum	65	56	60	65	65
	moyen	44	44	43	38	39
**Sexe (%)**	féminin	40	33	29	65	66
	masculin	60	67	71	35	34
**Profession (%)**	aide-soignant	0	10	1	49	53
	infirmier	0	44	41	32	32
	sage-femme	0	6	11	11	9
	médecin	70	26	24	5	3
	autre catégorie	30	13	23	3	3
**Fonction (%)**	vaccinateur	0	7	5	76	77
	gestionnaire PEV	50	22	22	8	6
	chargé communication	0	7	13	6	4
	chargé surveillance maladies	20	18	16	2	2
	logisticien	30	16	18	0	0,9
	autre fonction	0	29	25	8	9
**Statut administratif (%)**	contractuel de l’État	0	54	57	57	56
	fonds communautaire	0	0	5	27	27
	fonctionnaire	70	46	37	9	9
	collectivité locale	0	0	0	1	1
	autre statut	30	0	1	6	7
**Ancienneté de la formation PEV (%)**	non formé	50	21	27	54	56
	5 ans et plus	50	53	43	14	14
	moins de 5 ans	0	26	30	32	29
**Nature de la formation reçue (%)**	vaccination	50	71	65	48	44
	gestion vaccins	30	40	49	17	17
	surveillance maladies	50	53	50	17	16
	chaîne du froid	30	32	38	14	15
	maintenance	0	19	12	4	4
**Souhait de formation**	oui	100	97	100	98	98
**Nature de la formation souhaitée (%)**	vaccination	20	40	62	76	74
	gestion vaccins	60	63	57	66	69
	surveillance maladies	40	46	64	61	61
	chaîne du froid	60	60	52	58	61
	maintenance	50	60	57	47	47

DVL: Direction de la vaccination et logistique DDS: Directions départementales de la santé BZS: Bureaux de zone sanitaire

Sur les 3 893 acteurs recensés, 44% étaient formés dont 44% en vaccination, 17% en gestion des vaccins, 16% en surveillance des maladies, 15% en chaîne du froid et 4% en maintenance des équipements. Cinquante-six pour cent des acteurs n’étaient pas formés et 14% étaient formés depuis 5 ans ou plus. Nous avons donc estimé que 70% des acteurs avaient besoin de formation sur le PEV dont 100% à la Direction de la vaccination et logistique, 74% dans les directions départementales de la santé, 70% dans les bureaux de zone sanitaire (BZS) et 68% dans les centres périphériques. La proportion d'acteurs ayant déclaré avoir besoin de formation était de 98% dont 100% de la Direction de la vaccination et logistique, 97% des directions départementales de la santé, 100% des bureaux de zone sanitaire et 98% des centres périphériques. Les acteurs ont souhaité être formés en vaccination (74%), gestion des vaccins (69%), chaîne du froid (61%), surveillance (61%) et maintenance (47%).

### Profil et formation des acteurs par type de structure de soins

La vaccination était offerte dans 5 centres nationaux hospitalo-universitaires (CNHU), 6 centres hospitaliers départementaux (CHD), 30 hôpitaux de zone (HZ), 805 centres de santé (CS) et 58 structures privées (cabinets, cliniques). Les 3893 acteurs étaient répartis comme suit: centre national hospitalo-universitaire (0, 2%), centre hospitalier départemental (2%), hôpital de zone (3%), centre de santé (92%) et structures privées (3%). Dans les centres nationaux hospitalo-universitaires, c’était les infirmiers et les sages-femmes qui vaccinaient et ils étaient presque tous formés sur tous les aspects du PEV. Dans les centres hospitaliers départementaux et hôpitaux de zone, les acteurs appartenaient à toutes les catégories professionnelles avec une prédominance des infirmiers et une faible proportion des aides-soignants; toutes les fonctions étaient présentes et les besoins de formation étaient de 79% et 72%. Dans les centres de santé, c’était les aides-soignants (54%) qui vaccinaient et les besoins de formation étaient de 73% alors que dans les structures privées, c’était des infirmiers et les besoins de formation étaient de 52% (Tableau II).

**Tableau II:** Répartition et profil des acteurs de la vaccination par type de structure de soins, Bénin, 2020 Distribution and profile of immunization stakeholders according to type of health facility, Benin, 2020

		CNHU	CHD	Hôpitaux de zone	Centres de santé	Structures privées
		(n = 8)	(n = 76)	(n = 99)	(n = 3592)	(n = 118)
**Profession (%)**	aide-soignant	0	5	2	54	7
	infirmier	50	42	38	34	60
	sage-femme	50	17	16	7	17
	médecin	0	24	22	2	8
	autre catégorie	0	12	21	3	8
**Fonction (%)**	vaccinateur	100	20	12	81	72
	gestionnaire du PEV	0	21	20	6	2
	chargé communication	0	7	12	4	0
	chargé surveillance maladies	0	16	15	2	0
	logisticien	0	14	17	0	0
	autre fonction	0	22	23	7	26
**Ancienneté de la formation PEV (%)**	moins de 5 ans	88	21	28	27	48
	5 ans et plus	0	47	40	14	0
	non formé	12	32	32	59	52
**Nature de la formation reçue (%)**	vaccination	88	63	55	39	42
	gestion vaccins	88	36	46	21	44
	surveillance maladies	88	47	36	15	41
	chaîne du froid	75	29	45	9	21
	maintenance	88	17	38	6	31

CNHU: Centre national hospitalo-universitaire, CHD: Centre hospitalier départemental

### Profil et formation des acteurs selon la fonction au sein du PEV

Les fonctions de vaccinateur et chargé de communication étaient assurées par des aides-soignants (58% et 77%) alors que celles de gestionnaire du PEV, chargé de surveillance et logisticien étaient assurées par des infirmiers (70%, 65% et 77%). La majorité des vaccinateurs (54%), chargés de communication (78%), chargés de surveillance (57%) n’étaient pas formés sur le PEV et seuls 23% des chargés de communication, 29% des chargés de surveillance, 45% des vaccinateurs, 61% des gestionnaires PEV étaient formés dans leurs domaines. La proportion d'acteurs ayant besoin d’être formés était estimée à 71% pour les vaccinateurs, 54% pour les gestionnaires, 87% pour les chargés de communication, 79% pour les chargés de surveillance et 66% pour les logisticiens. La proportion d'acteurs ayant déclaré avoir besoin de formation était de 99% pour les vaccinateurs, 97% pour les gestionnaires PEV, 100% pour les chargés de communication, 84% pour les chargés de surveillance et 97% pour les logisticiens. Au moins 60% des vaccinateurs souhaitaient une formation en vaccination, gestion des vaccins et chaîne du froid; 2/3 des gestionnaires du PEV voulaient être formés en vaccination, gestion des vaccins, surveillance et chaîne de froid, et au moins 60% des logisticiens souhaitaient une formation en surveillance et en maintenance. Les chargés de surveillance (au moins 67%) et les chargés de communication (au moins 82%) souhaitaient une formation dans tous les domaines du PEV (Tableau III).

**Tableau III:** Répartition et profil des acteurs de la vaccination selon la fonction occupée, Bénin, 2020 Distribution and profile of immunization stakeholders according to function, Benin, 2020

		Vaccinateur	Gestionnaire PEV	Chargé Communication	Chargé Surveillance	Logisticien
		(n = 3013)	(n = 246)	(n = 171)	(n = 84)	(n = 35)
**Profession (%)**	aide-soignant	58	0,8	77	13	3
	infirmier	29	70	6	65	77
	sage-femme	10	8	4	4	3
	médecin	0,4	19	2	7	0
	autre catégorie	2	2	11	11	17
**Ancienneté de la formation PEV (%)**	moins de 5 ans	29	47	13	21	34
	5 ans et plus	17	24	9	22	57
	non formé	54	29	78	57	9
**Nature de la formation reçue (%)**	vaccination	45	61	23	28	80
	gestion vaccins	13	50	15	16	80
	surveillance maladies	15	39	4	29	51
	chaîne du froid	13	30	3	11	66
	maintenance	4	15	1	2	31
**Souhait de formation (%)**	oui	99	97	100	84	97
**Nature de la formation souhaitée (%)**	vaccination	75	70	95	69	29
	gestion vaccins	60	66	88	69	51
	surveillance maladies	51	67	88	67	60
	chaîne du froid	60	67	85	72	40
	maintenance	46	48	82	67	66

## Discussion

### Discussion de la méthodologie

Nos résultats sont représentatifs de la situation au Bénin en 2020, car il s'agit d'un recensement qui a combiné la consultation des dossiers administratifs et les entretiens téléphoniques. Il faut signaler que les multiples relances téléphoniques et le dispositif de vérification de la complétude des données collectées ont permis d'obtenir cet excellent taux de réponse de 100%.

### Discussion des résultats

La répartition des acteurs du PEV selon l’âge et le sexe est comparable à celle du personnel de santé en 2018 [6]. En effet, 35% des acteurs du PEV et 36% du personnel de santé avaient entre 35 et 44 ans. La prédominance du sexe féminin est plus marquée chez les acteurs du PEV que chez le personnel de santé (66% *versus* 52%) parce que le système de santé affecte plus de femmes aux activités de vaccination car les bénéficiaires sont les jeunes enfants et les femmes. La prédominance des aides-soignants dans la vaccination était observée au Bénin dans l'enquête SARA (Services Availability and Readiness Assessment) de 2018 [5]. Ceci est dû à l'insuffisance d'agents de santé qualifiés dans les centres de santé et au fait qu'au Bénin, ces aides-soignants qui ont comme diplôme le certificat d’études primaires élémentaires, sont reconnus et régis par le décret n° 2009-129 du 16 avril 2009, qui décrit leurs attributions, les modalités de leur recrutement et précise qu'ils devraient participer aux activités de soins préventifs et promotionnels [12]. C'est ce qui justifie et officialise leur implication dans le PEV où ils assurent surtout les fonctions de vaccinateur et de chargé de communication. Compte tenu de leur profil, il est important qu'ils soient formés sur le PEV et régulièrement supervisés. Le statut administratif des acteurs du PEV est similaire à celui du personnel de santé présenté dans le cadre organique 2018-2020 [5]. La grande majorité des acteurs de la vaccination exerce dans des centres périphériques (92%), ce qui est cohérent puisque ces centres sont les points d'entrée des soins de santé primaires.

Au Bénin, la majorité des acteurs de vaccination (70%) ont besoin d’être formés sur le PEV et plus de la moitié des vaccinateurs, chargés de communication et chargés de surveillance ne sont pas formés dans leurs domaines. Ils ne sont donc pas aptes à assurer efficacement leurs fonctions. Cette insuffisance concerne tous les niveaux de la pyramide sanitaire. Le besoin estimé de formation concerne 70% des acteurs alors qu'ils sont 98% à vouloir être formés. Ce besoin déclaré de formation est-il réel, ou reflète-t-il une simple envie d'aller en formation ? Néanmoins, il faut préciser que la plupart des domaines dans lesquels ils souhaitent être formés sont en lien avec leurs fonctions. Le besoin de formation ne semble pas être propre au Bénin. En effet, une étude réalisée en 2021 dans 9 pays africains indiquait aussi que 98, 3% des personnes interrogées ont déclaré avoir des défauts de compétences qui nécessiteraient des formations sur le PEV [2]. Les domaines les plus cités pour ce besoin de formation étaient la surveillance des maladies et la qualité des données, le financement durable de la vaccination, la surveillance des effets indésirables et la mobilisation communautaire.

Les dernières revues externes du PEV de certains pays francophones d'Afrique subsaharienne [3, 4, 9, 10, 11] font aussi état du niveau de formation du personnel de vaccination. Elles avaient révélé que d'une façon générale, la majorité des acteurs de la vaccination n’étaient ni qualifiés, ni formés sur le PEV. Ces informations étaient collectées surtout auprès des chefs des centres de vaccination. Bien que ces revues corroborent le « faible niveau de formation sur le PEV », aucune d'entre elles ne quantifie ni la proportion d'acteurs ayant besoin de formation sur le PEV, ni les domaines prioritaires pour leur formation.

## Conclusion

Cette étude qui est faite pour la première fois au Bénin, montre que la moitié des vaccinateurs ont un profil inadapté aux activités qui leur sont confiées. La Direction de la vaccination et logistique devrait intégrer cette réalité dans sa planification, et disposer d'un plan de renforcement de capacité de ces acteurs basé sur les résultats de cette étude. Elle devrait également mobiliser les ressources pour la mise en œuvre de ce plan, afin de garantir la qualité et la sûreté vaccinales et améliorer la performance du PEV au Bénin. Il est important qu'un accent particulier soit mis sur la formation et la supervision régulière des aides-soignants.

## Contribution Des Auteurs

Protocole: Aplogan, Agossadou, Palenfo

Collecte et traitement: Aplogan, Agossadou Palenfo

Analyse: Aplogan, Agossadou

Rédaction: Aplogan, Palenfo

Révision: Kaucley, Essoh, Ahawo

## Liens D'intérêts

Les auteurs ne déclarent aucun lien d'intérêt.
